# Exploration of Chlamydial Type III Secretion System Reconstitution in *Escherichia coli*

**DOI:** 10.1371/journal.pone.0050833

**Published:** 2012-12-11

**Authors:** Xiaofeng Bao, Wandy L. Beatty, Huizhou Fan

**Affiliations:** 1 Department of Pharmacology, Robert Wood Johnson Medical School, University of Medicine and Dentistry of New Jersey, Piscataway, New Jersey, United States of America; 2 Department of Pharmacology, School of Medicine, Nantong University, Nantong, People's Republic of China; 3 Department of Molecular Microbiology, Washington University School of Medicine, St. Louis, Missouri, United States of America; University of California Merced, United States of America

## Abstract

**Background:**

Type III secretion system is a virulent factor for many pathogens, and is thought to play multiple roles in the development cycle and pathogenesis of chlamydia, an important human pathogen. However, due to the obligate intracellular parasitical nature of chlamydiae and a lack of convenient genetic methodology for the organisms, very limited approaches are available to study the chlamydial type III secretion system. In this study, we explored the reconstitution of a chlamydial type III secretion in *Escherichia coli*.

**Results:**

We successfully cloned all 6 genomic DNA clusters of the chlamydial type III secretion system into three bacterial plasmids. 5 of the 6 clusters were found to direct mRNA synthesis from their own promoters in *Escherichia coli* transformed with the three plasmids. Cluster 5 failed to express mRNA using its own promoters. However, fusion of cluster 5 to cluster 6 resulted in the expression of cluster 5 mRNA. Although only two of the type III secretion system proteins were detected transformed *E. coli* due to limited antibody availability, type III secretion system-like structures were detected in ultrathin sections in a small proportion of transformed *E. coli*.

**Conclusions:**

We have successfully generated *E. coli* expressing all genes of the chlamydial type III secretion system. This serves as a foundation for optimal expression and assembly of the recombinant chlamydial type III secretion system, which may be extremely useful for the characterization of the chlamydial type III secretion system and for studying its role in chlamydial pathogenicity.

## Introduction

Gram-negative bacterial pathogens use the type III secretion (T3S) system (T3SS) to communicate with their eukaryotic host cells [1]. Upon physical contact of bacteria with host cells, bacterial cytosolic proteins referred to as T3S effectors are translocated to the eukaryotic cells through a needle-like structure referred to as “injectisome”. In addition to the needle connecting the bacterium and the eukaryotic cell, the injectisome consists of ring-like structures on the bacterial inner and outer membranes, and a pore-like structure made of proteins designated translocators on the target eukaryotic cell membrane.

The inner diameter of the T3SS needle is about 2.5 nm, permitting the passage of only unfolded proteins [2]. Emerging evidence suggests that prior to transportation many T3S effectors exist in partially unfolded conformation and are associated with T3SS protein chaperones, which keep the effectors from being degraded in the bacterial cytosol. Hydrolysis of ATP by the T3SS ATPase, located at the cytoplasmic interface of the basal body, causes the dissociation of T3S effectors from their chaperones. The energy provided by the ATPase is also used to (further) unfold the effectors, allowing them to enter and travel through the narrow needle [1], [3], [4], [5], [6].

Though the overall structure of the T3SS injectisome is highly alike among various organisms, T3S plays distinct roles in the pathogenesis of different infections. Pathogenic *Yersinia* spp secrete outer proteins designated Yops, which inhibit phagocytosis of macrophages and neutrophils, thus permitting their extracellular survival and replication [7]. In contrast, *Salmonella* spp use a T3SS encoded to mediate the uptake of bacterium into the host cell [8]. Inside the cell, another T3SS is activated, causing the rupture of *Salmonella*-containing vacuoles and the release of bacteria into the cytoplasm [9].

*Chlamydia* is an obligate intracellular bacterium that is responsible for or contributes to a number of human diseases including sexually transmitted infection, preventable blindness, respiratory tract infection, and arteriosclerosis [10], [11]. *Chlamydia* has a unique developmental cycle, which alternates between two cellular forms: the infectious but metabolically-inert elementary body (EB) and the vegetative but non-infectious reticulate body (RB) [12]. The developmental cycle starts with the attachment of an EB to the host cell plasma membrane. The EB is taken up into a vacuole designated an inclusion. Inside the inclusion, the EB develops into the dividing RB. After successive binary fissions, most RBs differentiate back into EBs before the completion of the developmental cycle and the release of chlamydiae from the host cell [13], [14].

Similar to most other Gram-negative pathogens, *Chlamydia* encodes a T3SS. The chlamydial T3SS (cT3SS) is thought to form projections on the chlamydial cell surface detected with electron microscopy [15], [16] . cT3SS appears to play multiple roles throughout the chlamydial developmental cycle [[17], [18] for review]. Thus, immediately upon cell entry, EBs secrete TARP, a T3S effector, into the cytoplasm. By recruiting actin to the site of EB internalization, secreted TARP enables the trafficking of the early chlamydial inclusions [19], [20], [21]. In later stage points, RBs secrete a variety of protein via the T3SS to the inclusion membrane or host cell cytoplasm [22], [23], [24], [25], [26], [27]. The importance of chlamydial T3S has been implicated by abnormal intracellular development as results of inhibition of the T3S or the effectors [24], [28], [29].

Investigation of chlamydial T3S, to a large degree, has relied on the use of small T3S inhibitors [24], [28], [29] and/or surrogate T3SSs [30], [31], [32] because of the obligate intracellular nature of the organism and a lack of a convenient genetic manipulation system. While these approaches have yielded valuable information for the cT3SS, they both have limitations. Therefore, even though small T3SS inhibitors demonstrate specificity in targeting T3S in free-living bacteria, interpretation of their effects on *Chlamydia* is complicated by their significant toxicity to the host cell and by the reversal of the effects on both the host cell and cT3S with iron ions [33]. A surrogate system may not operate the same way as the cT3SS. For example, the chlamydial T3S chaperones Scc2 and Scc3 fail to fully complement the deficiency of its *Yersinia* homolog sycD [30]. In addition, the chlamydial T3S effectors CopD and Pkn5 cannot be secreted by the T3SS encoded by the pathogenic island-1 although they can be translocated by the one encoded by the pathogenic island-2 [32]. The limited availability of research tools calls for the development of new approaches to studying chlamydial T3S. The aim of this work is to explore the possibility of reconstituting cT3SS in *E. coli.* We cloned all the 6 cT3SS-encoding clusters into three plasmids, and derived *E. coli* that expresses genes from all the 6 clusters concurrently. We further present evidence for the assembly of T3SS proteins into a hypothetical T3SS structure despite low efficiency.

## Materials and Methods

### Strains

*C. trachomatis* serovar D (strain UW-3/Cx) and serovar L2 (strain 434/bu) were purchased from ATCC. Transformation-competent *E. coli* stbl2 was purchased from Stratagen.

### Reagents

Primers used for vector construction (Table S1), DNA sequencing (Table S2) and/or reverse-transcriptase PCR (RT-PCR) (Table S3) were custom-synthesized at Sigma-Aldrich. PfuUltra II Fusion HS DNA polymerase was purchased from Stratagen. DNA endorestriction enzymes, T4 DNA ligase and Taq DNA polymerase were purchased from New England Biolabs. The TaqMan RT-PCR kit was purchased from Applied Biosystems. Monoclonal anti-Flag M2 antibody was purchased from Sigma-Aldrich. Rabbit polyclonal anti-TARP was generously provided by Dr. Ted Hackstadt (Rocky Mountain Laboratories, National Institutes of Health). All other primary antibodies were generous gifts from Dr. Guangming Zhong (University of Texas Health Sciences Center at San Antonio).

### Molecular cloning

**Figure 1:**
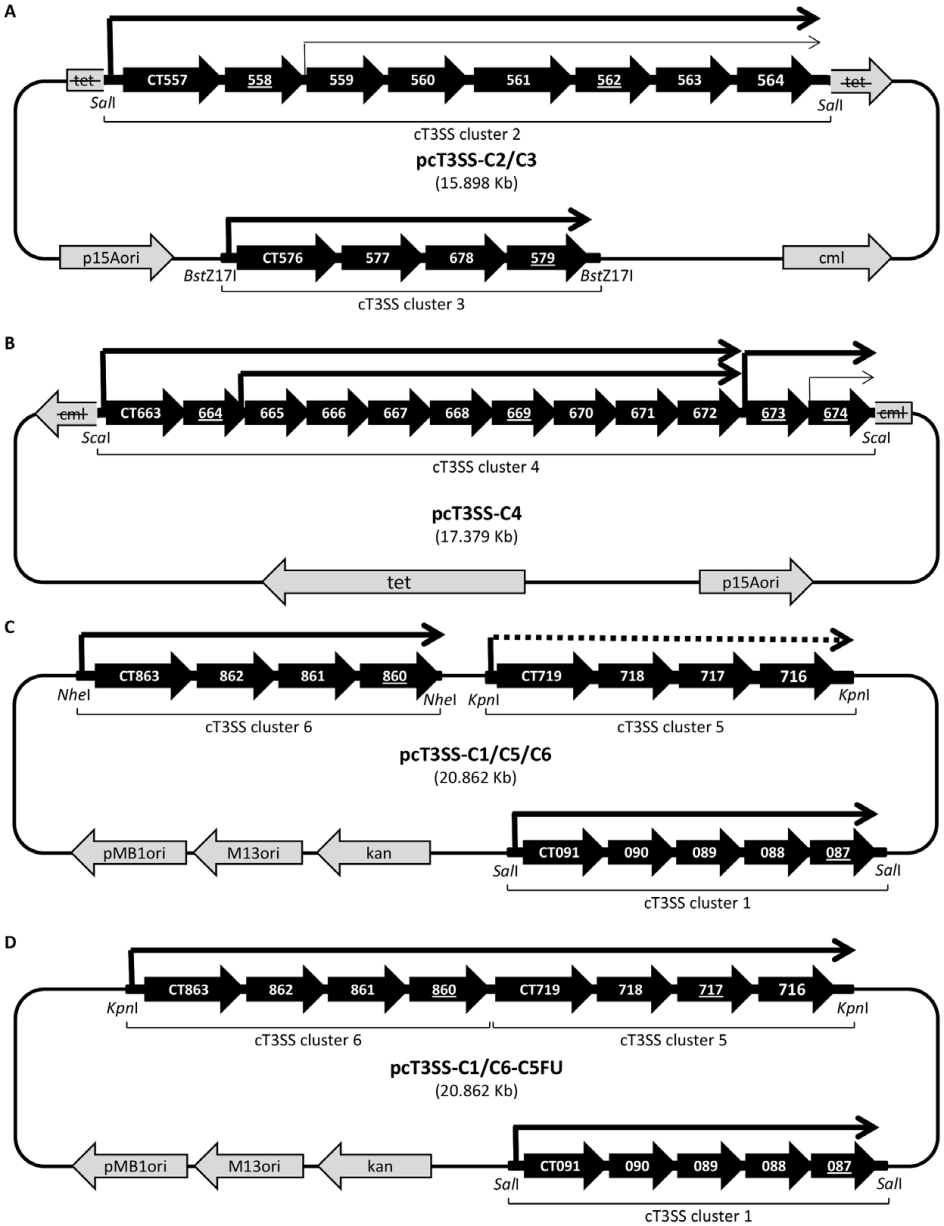
cT3SS expression vectors. Plasmids in panels A–D contain different cT3SS clusters. The tetracycline-resistance gene (tet) and chloramphenicol-resistance gene (cml) are inactivated in A and B, respectively, as results of the insertion of cT3SS fragments. Thick black arrows show cT3SS genes, as numbered in the *C. trachomatis* serovar D genome. Genes of which mRNA was detected by RT-PCR are underlined. Lengths of genes are not in scale. Line arrows signify locations of promoters and direction of transcription in operons as previously established [39]. Dotted line in C indicates no detectable transcription from the promoter shown. Transcription from two internal promoters in panels A and B is unlikely to occur in *E. coli* (shown in thinner line arrows). The activities of these two promoters were not specifically examined in this study. Endorestriction sites used for cloning are shown in italics. The orientations of the cT3SS clusters in the plasmids were not determined and are shown arbitrarily.

A total of 19 plasmids, 12 for cT3SS and 7 for cT3S effectors, were constructed for this study (Table 1). Four final plasmids that were used for expression and characterization of cT3SS expression in *E. coli* are highlighted in Table 1 and shown in Fig. 1. In general, DNA fragments of cT3SS clusters and T3S effector genes were amplified from the genomic DNA of *C. trachomatis* D using the high fidelity PfuUltra II Fusion HS DNA polymerase for PCR and primers carrying appropriate endorestriction enzyme cutting sites. PCR products were gel-purified, digested with an endorestriction enzyme and cloned into an appropriate vector. Sequence authenticity of inserts was confirmed by customer DNA sequencing at Genewiz. Specifically, cluster 2 (nt 628831-636379) was directly cloned into the *Sal*I site of pACYC184 to yield plasmid pcT3SS-C2 (Table 1). Cluster 3 (nt 648613-652718) was amplified with primers each carrying a *Bst*Z17I site and a *Sal*I site. The PCR product was first cloned into the *Sal*I site of pBluescript II KS+, and then copy-pasted into the *Bst*Z17I site of pcT3SS-C2 to yield pcT3SS-C2/C3 (Table 1 and Fig. 1A). Cluster 4 (nt 760280-773414) was first amplified as two separate fragments (fC4a of nt 76280-767358 with a *Sca*I site added to the 5′ end, and fC4b of nt 767245-773414 with a *Sca*I site added to the 3′ end) because the 13,135 bp full-length exceeded the amplification capacity of the DNA polymerase. fC4a and fC4b were joined by the T4 ligase following digestion *Mfe*I. The ligation product was further digested with *Sca*I and cloned into the *Sca*I site of the pACYC184 plasmid to yield pcT3SS-C4 (Table 1 and Fig. 1B). Cluster 6 (nt 1017829-1012656 in the genomic DNA) was cloned into the *Nhe*I site of pBAD18-kan to yield pcT3SS-C6 (Table 1). Cluster 5 (nt 831936-828219) was then cloned into the *Kpn*I site of pcT3SS-C6 to yield pcT3SS-C5/C6 (Table 1). Cluster 1 (nt 106660-99851) was first cloned into pBluescript II KS+ using the *Sal*I site and then moved into pcT3SS-C5/C6 to yield pcT3SS-C1/C5/C6 (Table 1 and Fig. 1C). pcT3SS-C1/C5-C6FU represented a variant of pcT3SS-C1/C5/C6. To construct pcT3SS-C1/C6-C5FU, cluster 6 was fused to nt 831714-828219 of cluster 5 using two-step PCR. The final PCR product was cloned into pBAD18-kan plasmid to yield pcT3SS-C6-C5FU. Cluster 1 was transferred from cloned into pcT3SS-C5-C6FU, as described above, to yield pcT3SS-C1/C6-C5FU (Table 1 and Fig. 1D).

**Table 1:** Vector information.

Plasmid	Description
pBluescript II-C1	pBluescript II KS+ containing cluster 1, sequenced
pcT3SS-C2	pACYC184 containing cluster 2, sequenced
pBluescript II-C3	pBluescript II KS+ containing cluster 3, sequenced
**pcT3SS-C2/C3**	pACYC184 containing cluster 2 and cluster 3 from pBluescript II-C3
pBluescript II-C4a	pBluescript II KS+ containing cluster 4a, sequenced
pBluescript II-C4b	pBluescript II KS+ containing cluster 4b, sequenced
**pcT3SS-C4**	pACYC184 containing cluster 4 from pBluescript II-C4a and pBluescript II-C4b
pcT3SS-C6	pBAD18-Kan containing cluster 6, sequenced
pcT3SS-C5/C6	pBAD18-Kan containing cluster 6 and cluster 5, sequenced
**pcT3SS-C1/C5/C6**	pBAD18-Kan containing cluster 1 (from pBluescript II-C1), cluster 5 and cluster 6
pcT3SS-C6-C5FU	pBAD18-Kan containing C6-C5FU, sequenced
**pcT3SS-C1/C6-C5FU**	pBAD18-Kan containing cluster 1 and C6-C5FU
pRK5_TARP-Flag	TARP coding sequence was inserted between NdeI and SalI of pRK5-cFlag
**pBAD24_TARP-Flag**	*C*-Flag-TARP was cutted out by *Nde*I & *Hind*III from pRK5_TARP-Flag and pasted into pBAD24
**pBAD24_IncA-Flag**	IncA coding sequence replaced TARP coding sequence of pBAD24_TARP-Flag
**pBAD24_IncD-Flag**	IncD coding sequence replaced TARP coding sequence of pBAD24_TARP-Flag
**pBAD24_IncG-Flag**	IncG coding sequence replaced TARP coding sequence of pBAD24_TARP-Flag
**pBAD24_CT813-Flag**	CT813 coding sequence replaced TARP coding sequence of pBAD24_TARP-Flag
**pBAD24_TARP**	TARP ORF placed between *Nde*I and *Sal*I sites of pBAD24

Vectors used for experiments to determine cT3SS expression and T3S are shown in boldface.

Expression vectors for five T3S effectors, TARP, IncA, IncD, IncG and CT813 were constructed with similar strategies (Table 1). The coding sequence of TARP was first cloned into pRK5-Flag using *Nde*I and *Sal*I digestion; the TARP-Flag sequence was then copy-pasted into pBAD24 between the *Nde*I and *Hind*III sites. To generate expression vectors for the other T3S effectors, the TARP coding sequence in the pBAD24_TARP-Flag vector was released by *Nde*I and *Sal*I double digestion and replaced by the coding sequences of IncA, IncD, IncG or CT813. Similar to pBAD24_TARP-Flag, the additional T3S vectors carried an in-frame Flag tag-coding sequence at the 3′-terminus. Sequence authenticity was confirmed by sequencing. Finally, to obtain an expression vector for non-tagged TARP, its open reading frame (including the stop codon) was cloned into pBAD24 between the *Nde*I and *Sal*I sites.

### Reverse transcriptase PCR (RT-PCR)

*E. coli* was cotransformed with the three cT3SS expression vectors and selected on an LB Agar plate containing chloramphenicol, tetracycline and kanamycin. LB broth was inoculated with a colony confirmed by PCR to contain all three plasmids, and cultured overnight in a shaker incubator at 37°C. A 250 µl overnight culture was used to inoculate 50 ml fresh LB in a 250 ml culture flask. The bacteria were allowed to grow in a shaker incubator at 37°C. When the OD_600_ of the culture reached 0.4∼0.5, bacteria were collected by centrifugation, and resuspended with 500 µl lysis buffer (0.5% SDS, 20 mM sodium acetate and 10 mM EDTA in DEPC-treatedwater, pH 4.0). Total RNA was extracted first with H_2_O-saturated phenol and then with chloroform, and precipitated with ethanol precipitation. Contaminating DNA was removed by two rounds of digestion with DNase I, which was then removed by a DNase-inactivating reagent (Ambion) [34]. cDNA synthesis and amplification were carried out using a TagMan RT-PCR kit.

### Detection of T3S in *E. coli*

*E. coli* was cotransformed with the three cT3SS expression vectors and an expression vector for a cT3S effector (TARP-Flag, IncA-Flag, IncD-Flag, IncG-Flag or CT813-Flag) and selected on an LB Agar plate containing chloramphenicol, tetracycline, kanamycin and ampicillin. LB broth, supplemented with 0.2% arabinose, was inoculated with a colony confirmed by PCR to contain all four plasmids, and cultured overnight in a shaker incubator at 37°C. The overnight culture was diluted with Dulbecco's modified Eagle's medium (DMEM) to 0.3 OD_600_. The dilution was divided into 1.6 ml aliquots, to which EGTA (final concentration: 2 mM), heat-inactivated fetal bovine serum (FBS, final concentration: 3%), both or neither, was added. The aliquots were incubated in a 5% CO_2_ incubator at 37°C for 6 h. A 1.0 ml sample of each culture was centrifuged at 20,000 g at 4°C for 3 min. The resulting pellet was dissolved in 200 µl SDS-PAGE sample buffer, and subjected to sonication [35]. The supernatant was centrifuged again and filtered through a 0.2 µM filter (Millipore) to remove any residual bacteria. 800 µl of the filtration was mixed with 200 µl ice-cold 100% (v/v) trichloroacetic acid (TCA). The mixture was incubated on ice for 1 h and centrifuged at 20,000 g at 4°C for 30 min. Proteins in the pellet were washed with 1 ml ice-cold acetone twice, air dried, re-dissolved in 10 µl SDS-PAGE buffer and resolved by SDS-PAGE along with a 5 µl sample of bacterial extract. Flag-tagged T3S effectors were detected by western blotting using the M2 antibody [36].

*E. coli* was also cotransformed with three cT3SS expression vectors and an expression vector for non-tagged TARP, and selected on an LB Agar plate containing chloramphenicol, tetracycline, kanamycin and ampicillin. LB broth, supplemented with 0.2% arabinose, was inoculated with a colony confirmed by PCR to contain all four plasmids, and cultured overnight in a shaker incubator at 37°C. The overnight culture was subjected to treatment described above. Alternatively, bacteria were collected by centrifugation at 20,000 g for 30 min and were then resuspended in phosphate-buffered saline (PBS) to yield 0.6 OD_600_. Two 1.6 ml aliquots of the suspension were generated. CaCl_2_ was added to one of the aliquots (final concentration of CaCl_2_: 2 mM). The aliquots were incubated at 37°C for 30 min and then centrifuged. Protein in the supernatant was precipitated by TCA and subjected to western blotting with an anti-TARP antibody.

### Preparation of EBs

Highly purified EBs were prepared following a protocol of Caldwell et al [37] with modifications. Near-confluent HeLa cell monolayers were inoculated with a *C. trachomatis* L2 stock at the multiplicity of 1 inclusion-forming unit per cell, cultured with DMEM containing 10% fetal bovine serum (FBS), 20 µg/ml gentamycin and 1 µg/ml cycloheximide at 37°C for 40 h. Cells were removed from the plates using a Cell Lifter. All steps from this point onward were performed either on ice or at 4°C. Cells were collected by centrifugation at 35,000 g using a Sorvall SS34 rotor, resuspended in sucrose-phosphate-glutamate buffer (SPG) and disrupted by sonication. Cell nuclei were spun down by centrifugation at 500 g for 10 min. The chlamydial organisms in the supernatant were then separated from cell organelles by ultracentrifugation through 8 ml of 35% (vol/vol) RenoCal at 43,000 g for 60 min using an SW28 Beckman rotor. EBs, RBs and remaining host components in the pellet were resuspended and further separated from each other by ultracentrifugation through 40%/44%/52% RenoCal at 50,000 g for 90 min using an SW28 rotor. EBs were collected from the 44%–52% interface, diluted in SPG [37], centrifuged at 35,000 g for 10 min, washed . Following two additional washes with SPG, EBs were resuspended in appropriate buffer for secretion experiments.

### Detection of TARP secretion from EBs

Detection of TARP secretion from EBs were carried out following published procedures [38] with the modification that freshly purified EBs were immediately used for experiments. EBs were resuspended in PBS or PBS containing 2 mM CaCl_2_. 55 µl EB suspensions in PBS or PBS containing 2 mM CaCl_2_ was incubated at 37°C for 30 min, and centrifuged at 20,000 g for 30 min. A 40 µl sample of the supernatant was transferred into another tube, and centrifuged again. A 25 µl sample of the supernatant from the second centrifugation was collected for western blotting. Each assay contained 4×10^8^ inclusion-forming units as determined retrospectively.

### Ultrathin section transmission electron microscopy

For analysis at the ultrastructural level, bacteria were fixed in 2% paraformaldehyde/2.5% glutaraldehyde/phosphate buffer, pH 7.2 for 1 hr at room temperature. Samples were washed in phosphate buffer and postfixed in 1% osmium tetroxide (Polysciences Inc., Warrington, PA) for 1 hr. Following extensive washing in dH_2_0, samples were *en bloc* stained with 1% aqueous uranyl acetate (Ted Pella Inc., Redding, CA) for 1 hr. Samples were then dehydrated in a graded series of ethanol and embedded in Eponate 12 resin (Ted Pella Inc.). Sections of 90 nm were cut with a Leica Ultracut UCT ultramicrotome (Leica Microsystems Inc., Bannockburn, IL), stained with uranyl acetate and lead citrate, and viewed on a JEOL 1200 EX transmission electron microscope (JEOL USA Inc., Peabody, MA).

## Results

cT3SS genes are organized into 6 clusters and 10 operons in the chlamydial genome [39]. We initially constructed 3 plasmids carrying the 6 clusters. The first two plasmids were both derived from pACYC184: one, designated pcT3SS-C2/C3, contained clusters 2 and 3, of 7,549 bp and 4,106 bp, respectively (Fig. 1A); the other, designated pcT3SS-C4, contained a single but large (13,135 bp) cluster 4 (Fig. 1B). The third plasmid, designated pcT3SS-C1/C5/C6, was a pBAD18-kan derivative, which contained the remaining three clusters of cT3SS: clusters 1, 5 and 6, of 5,174 bp, 3,718 bp and 6,810 bp, respectively (Fig. 1C). All cloned clusters carried their native promoters which have been previously identified [39].

**Figure 2:**
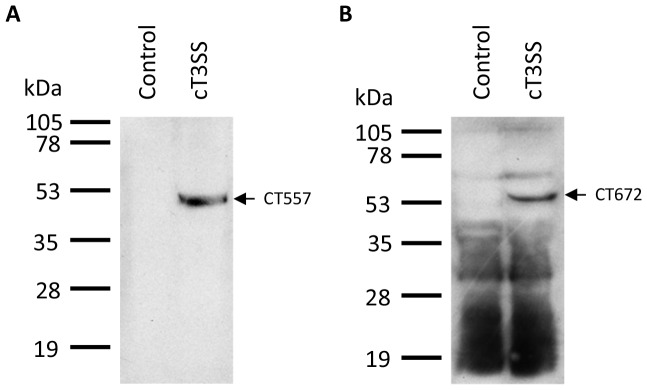
Detection of CT557 and CT672 in *E. coli* transformed with pcT3SS-C1/C5/C6, pcT3SS-C2/C3 and pcT3SS-C4 by western blotting. Control bacteria were transformed with empty pACYC184 and pBAD18 plasmids.

*E. coli* was co-transformed with the three plasmids and selected with three antibiotics, chloramphenicol, tetracycline and kanamycin, or with empty pACYC184 and pBAD18. Bacterial extracts were subjected to western blotting with antibodies for each protein of the cT3SS [40]. Among the 37 antibodies, only anti-CT557 and anti-CT672 each detected a protein specifically associated with cT3SS plasmid transformation (Fig. 2), whereas all other antibodies failed to demonstrate specificity in western blotting. The lack of a signal in the control vector-transformed *E. coli* suggests that the single protein band detected in the cT3SS-transformed bacteria shown in Fig. 2A was CT557. This was further supported by the fact that the protein band had a 49 kDa molecular mass predicted for CT557. On the other hand, anti-CT672 detected numerous protein bands in both cT3SS-transformed *E. coli* and control bacteria, in addition the protein band seen only in cT3SS-transformed bacteria which migrated as a 55 kDa protein (Fig. 2B). CT672 had a predicted 41 kDa molecular mass. Thus, CT672 appears to have a slower-than-expected gel mobility, similar deviations of actual gel mobility from the expected mobility based on the molecular mass have been documented for other proteins by us [41] and others [42].

**Figure 3:**
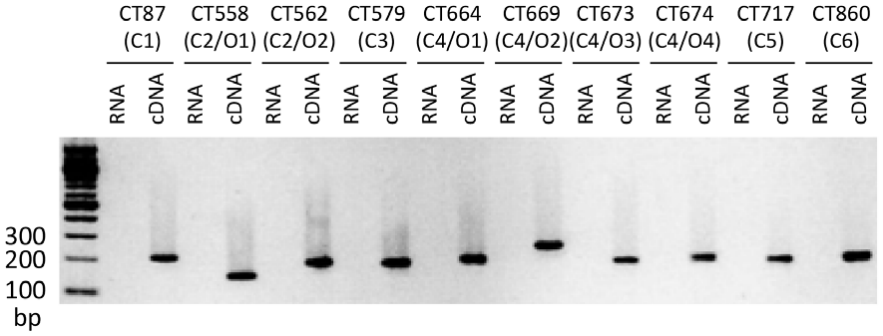
RT-PCR analysis of cT3SS gene transcription in *E. coli* transformed with pcT3SS-C1/C6-C5FU, pcT3SS-C2/C3 and pcT3SS-C4. Cluster (C) numbers and operon (O) numbers are shown. Note amplification occurred only in reaction for which cDNA but not RNA was used as template.

In the absence of evidence of protein expression from all clusters, we used RT-PCR to determine the expression of mRNA of cT3SS clusters in the transformed bacteria. We detected mRNA encoded by clusters 1–4 and 6 but not cluster 5 (data not shown). The absence of mRNA expression from cluster 5 is consistent with previous analysis demonstrating a lack of recognition of the single promoter of this cluster by *E. coli* [39]. To allow the expression of CT716–719 encoded by cluster 5, we removed the Rho-independent transcription termination signals of cluster 6 and the promoter of cluster 5 genes in pcT3SS-C1/C5/C6, thereby generating the pcT3SS-C1/C6-C5FU plasmid (Fig. 1D). The conversion of pcT3SS-C1/C5/C6 into pcT3SS-C1/C6-C5FU enabled the expression of cluster 5. Accordingly, *E. coli* transformed with pcT3SS-C1/C6-C5Fu, pcT3SS-C2/C3 and pcT3SS-C4 produced mRNA from all 6 clusters of cT3SS, as demonstrated by RT-PCR (Fig. 3). For clusters 2 and 4, multiple pairs of primers were used in RT-PCR, allowing for the detection of mRNA of all cT3SS operons. The successful amplification of cDNA with each and every primer pair tested (Fig. 3) suggests that all cT3SS genes are transcribed in *E. coli*.

In light of the fact that cluster 5 mRNA could be expressed when cluster 5 was fused to cluster 6 but not when cluster 5 existed as an independent operon, we repeated western blotting for bacteria transformed with pcT3SS-C1/C6-C5FU, pcT3SS-C2/C3 and pcT3SS-C4 and for control bacterial using antibodies for cluster 5 proteins. However, these antibodies failed again to detect a protein specifically associated with cT3SS transformation (data not shown).

**Figure 4:**
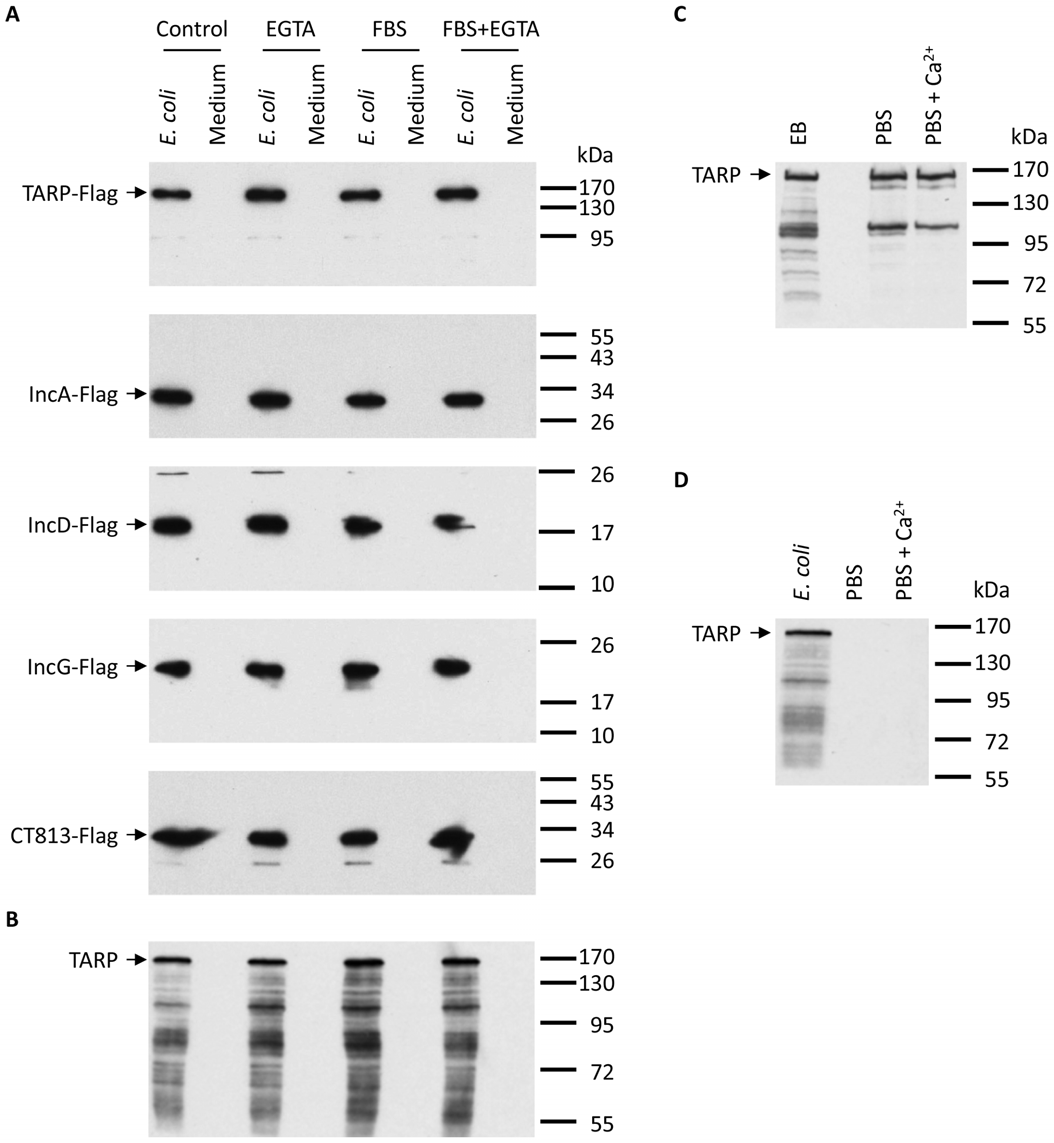
Lack of secretion of cT3S effectors in cT3SS plasmids-transformed *E. coli.* (A) Secretion of Flag-tagged T3S effectors from *E. coli* could not be induced by using DMEM (medium) containing the calcium-depleting reagent EGTA and/or FBS for 6 h. An anti-Flag antibody was used to detect the C-terminally tagged cT3S effectors. (B) Secretion of non-tagged TARP from *E. coli* was not induced under the same conditions as in (A). (C) TARP secretion from EBs suspended in PBS and PBS containing Ca^2+^. (D) Secretion of non-tagged TARP from *E. coli* was not detected using PBS or PBS containing Ca^2+^.

We next transformed *E. coli* with the three cT3SS expression vectors (pcT3SS-C1/C6-C5Fu, pcT3SS-C2/C3 and pcT3SS-C4) for all 6 expressible clusters and an expression vector for a cT3S effector carrying a C-terminal Flag tag (TARP-Flag, IncA-Flag, IncD-Flag, IncG-Flag or CT813-Flag), and determined if the effectors can be secreted from the cytoplasm into culture medium. Bacteria were exposed to conditions similar to those that induced T3S in enterobateria [43]. However, western blotting analysis failed to detect the presence of any of the three cT3S effectors in the medium under any of the conditions (Fig. 4A). Furthermore, no TARP secretion was detected under these conditions in *E. coli* contranformed with the three cT3SS expression vectors and an expression vector for non-tagged TARP (Fig. 4B).

Our inability to detect secretion of cT3S effector from T3SS-expressing *E. coli* prompted us to question the sensitivity of our detection technique. However, secretion of TARP were readily detected in EBs incubated in PBS (Fig. 4C) as previously demonstrated [38]. Similar level of TARP secretion was detected with PBS containing Ca2^+^. In contrast, no TARP secretion from the cT3SS-expressing bacteria was detected in PBS or PBS containing Ca2^+^ (Fig. 4D). Taken together, data presented in Fig. 4 suggest that T3S in the recombinant bacteria either did not occur or was extremely inefficient.

**Figure 5:**
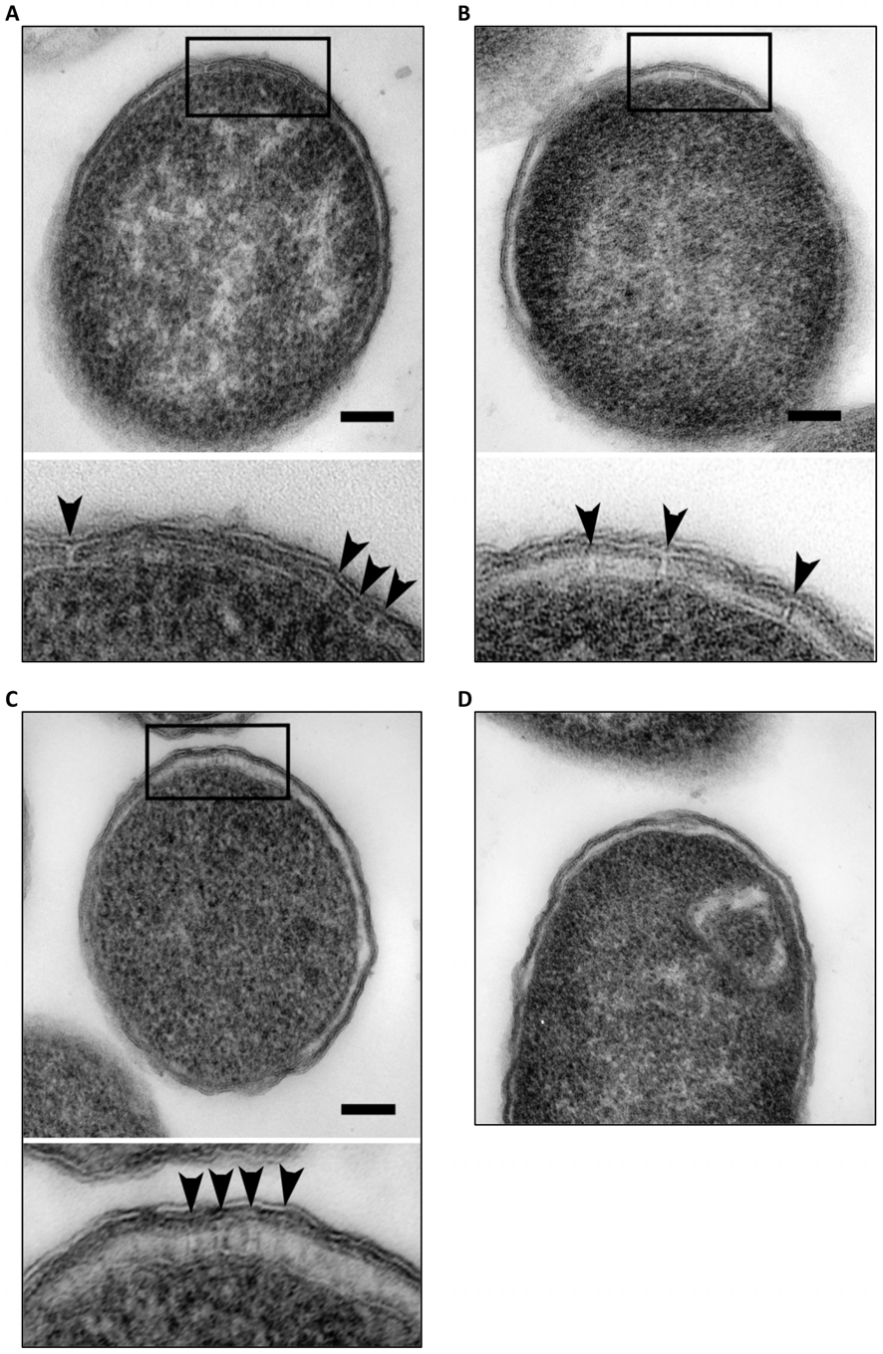
T3SS-like structures in *E. coli* transformed with cT3SS plasmids. Ultrathin section transmission electron microscopy revealed the presence of channel-like structures between the cytoplasmic membrane and outer membrane in *E. coli* transformed with pcT3SS-C1/C6-C5FU, pcT3SS-C2/C3 and pcT3SS-C4 (A–C). T3SS-like structures are shown more clearly in the enlarged images and indicated by arrowheads. These structures were not evident in ultrathin sections of control bacteria transformed with empty pACYC184 and pBAD18 plasmids (D). Scale bar is equal to 100 nm.

We used ultrathin section transmission electron microscopy to visualize if cT3SS protein expression would results in the formation of a T3SS-like structure(s) in bacterial membranes of cT3SS-expressing *E. coli.* Interestingly, we detected channel-like structures between the cytoplasm membrane and the outer membrane in a small proportion of transformed *E. coli* (Fig. 5A–C), whereas such structures were never detected in control bacteria transformed with control empty pACYC184 and pBAD18 (Fig. 5D), suggesting that the structures were recombinant cT3SS.

## Discussion

In the absence of a convenient tool to study cT3SS, we explored the reconstitution of cT3SS in *E. coli.* We were able to clone all 6 cT3SS cluster into 3 vectors, and co-transform them and a cT3S effector vector into *E. coli.* Whereas the expression of the effectors in *E. coli* was expected to be driven by a promoter in the vector, expression of genes in the cloned cT3SS clusters should be driven by their own promoters. We detected the expression of 2 cT3SS proteins, CT557 and CT672 in transformed *E. coli* (Fig. 2). Our inability to analyze the expression of the remaining proteins was due to high background of antibodies against chlamydial proteins. While these antibodies are highly useful for analyzing chlamydial protein expression in chlamydiae [27], [44], their usefulness in analyzing recombinant protein expression in *E. coli* is limited since the immunogens used to generate the antibodies were recombinant proteins carrying *E. coli* protein contaminants. Although only two of the 37 proteins encoded by the cT3SS clusters could be detected, the remaining 35 proteins were likely to be expressed in *E. coli* since we detected the expression of mRNAs encoded by all 10 operons in the 6 cT3SS clusters (Fig. 3) and the detection of T3SS-like structures in bacteria transformed with cT3SS expression vectors (Fig. 4).

Previous analyses have shown that promoters for 7 of the 10 cT3SS operons are active in *E. coli* [39]. The absence of the CT717 mRNA expression in *E. coli* transformed with pcT3SS-C1/C5/C6 in which cluster 5 is led by its own promoter (plus pcT3SS-C2/C3 and pcT3SS-C4) demonstrated by our RT-PCR analysis confirms non-recognition of the sole promoter of cluster 5 by the *E. coli* transcriptional machinery as previously reported [39]. The other two promoters that failed to function in *E. coli* in that study [39] were an internal promoters upstream of CT559 in clusters 2 and the second internal promoter upstream of CT674. Thus, the mRNAs for CT562 and CT674 detected in Fig. 3 are likely to originate from the transcripts of the primary operons rather than the secondary operons.

Despite of the evidence for cT3SS expression in *E. coli*, secretion in the transformed bacteria was undetectable for any of the five cT3S effectors analyzed (Fig. 4A,B,D). The reason for these negative findings may be multi-factorial. First, even though RT-PCR data suggest that all cT3SS genes are transcribed, their expression levels may not be high enough to allow for the production of enough cT3SS apparatuses in the fast-growing *E. coli* to efficiently secrete effectors since the heterologous expression still relied on the native cT3SS promoters, some which may function inefficiently in the enterobacterium. Second, the efficiency of biosynthesis of some cT3SS proteins might be low due to possible rare codon use in *E. coli*, which limited the formation of recombinant cT3SS, even if transcription occurred efficiently. Third, the epitope tag added to the C-terminus of the effectors may interfere with secretion even though the secretion signal is known to reside within the N-terminus [1], [6], [22]. Fourth, assembly of cT3SS proteins into a secretion apparatus in *E. coli*, an unnatural environment for chlamydial proteins, may also be insufficient. Finally, the recombinant cT3SS may not respond to the experimental conditions for secretion induction in *E. coli* regardless whether or not these conditions generate signals for secretion in chlamydiae.

Channel-like structures were detected between the cytoplasm membrane and outer membrane in a small number of bacteria carrying the entire 6 cT3SS clusters using ultrathin section transmission electron microscopy (Fig. 5). These structures were never found in control bacteria transformed with empty vectors. These findings indicate that the structures were formed by cT3SS proteins albeit at low efficiency. Nevertheless, in the absence of evidence for the secretion of cT3S effectors, it is not absolutely certain whether these channels are complete or incomplete T3SS, if at all. Demonstration of the existence of cT3SS proteins in the channels will be essential to establish the channels' identities. Unfortunately, the anti-CT557 antibody that we used is unlikely to be useful for characterization of the putative T3SS-like structures using immunoelectronic microscopy because it lacks the capacity to specifically detect signals in cT3SS-expressing *E. coli* in an immunofluorescence assay (data not shown), despite its high specificity shown in western blotting. Even if another antibody recognizes CT557 in immunostaining, it might have no value for analyzing cT3SS structures because CT557 is predicted to be a dihydrolipoamide dehydrogenase, an unrelated homolog of which the localization cannot be predicted. Furthermore, although CT672 is a predicted cT3SS apparatus protein, the strong nonspecific binding activity of the anti-CT672 (Fig. 2B) makes it worthless for immunoelectronic microscopy. For these considerations, we are performing epitope tagging for proteins predicted to be in the cT3SS structure apparatus to characterize the cT3SS-like structures in recombinant *E. coli* cells.

## Supporting Information

Table S1 **Primers for vector construction.** Chlamydial genomic DNA sequences are shown in lower cases. Sequences in upper cases were added to create enzyme-cutting sites (non-italics) and to facilitate digestion (italics).
(XLS)

Table S2 **Sequencing primers.** Number denotes position of primer's first base in the *C. trachomatis* D genome (accession #: NC_000117).
(XLS)

Table S3 **Primers for reverse-transcription PCR.** N/A: not applicable.
(XLS)
